# Lipidomic Signatures of Changes in Adiposity: A Large Prospective Study of 5849 Adults from the Australian Diabetes, Obesity and Lifestyle Study

**DOI:** 10.3390/metabo11090646

**Published:** 2021-09-21

**Authors:** Habtamu B. Beyene, Gavriel Olshansky, Corey Giles, Kevin Huynh, Michelle Cinel, Natalie A. Mellett, Adam Alexander T. Smith, Jonathan E. Shaw, Dianna J. Magliano, Peter J. Meikle

**Affiliations:** 1 Metabolomics Laboratory, Baker Heart and Diabetes Institute, Melbourne, VIC 3004, Australia; habtamu.beyene@baker.edu.au (H.B.B.); Gavriel.Olshansky@baker.edu.au (G.O.); Corey.Giles@baker.edu.au (C.G.); Kevin.Huynh@baker.edu.au (K.H.); michelle.cinel@baker.edu.au (M.C.); Natalie.Mellett@baker.edu.au (N.A.M.); alexander.smith@baker.edu.au (A.A.T.S.); 2 Faculty of Medicine, Nursing and Health Sciences, Monash University, Melbourne, VIC 3004, Australia; 3 School of Public Health and Preventive Medicine, Monash University, Melbourne, VIC 3004, Australia; Jonathan.Shaw@baker.edu.au (J.E.S.); Dianna.Magliano@baker.edu.au (D.J.M.); 4 Baker Heart and Diabetes Institute, Melbourne, VIC 3004, Australia

**Keywords:** plasma lipidomics, change in WC, change in BMI, metabolic scores

## Abstract

Lipid metabolism is tightly linked to adiposity. Comprehensive lipidomic profiling offers new insights into the dysregulation of lipid metabolism in relation to weight gain. Here, we investigated the relationship of the human plasma lipidome and changes in waist circumference (WC) and body mass index (BMI). Adults (2653 men and 3196 women), 25–95 years old who attended the baseline survey of the Australian Diabetes, Obesity and Lifestyle Study (AusDiab) and the 5-year follow-up were enrolled. A targeted lipidomic approach was used to quantify 706 distinct molecular lipid species in the plasma samples. Multiple linear regression models were used to examine the relationship between the baseline lipidomic profile and changes in WC and BMI. Metabolic scores for change in WC were generated using a ridge regression model. Alkyl-diacylglycerol such as TG(O-50:2) [NL-18:1] displayed the strongest association with change in WC (β-coefficient = 0.125 cm increment per SD increment in baseline lipid level, *p* = 2.78 × 10^−11^. Many lipid species containing linoleate (18:2) fatty acids were negatively associated with both WC and BMI gain. Compared to traditional models, multivariate models containing lipid species identify individuals at a greater risk of gaining WC: top quintile relative to bottom quintile (odds ratio, 95% CI = 5.4, 3.8–6.6 for women and 2.3, 1.7–3.0 for men). Our findings define metabolic profiles that characterize individuals at risk of weight gain or WC increase and provide important insight into the biological role of lipids in obesity.

## 1. Introduction

The prevalence of obesity has increased dramatically over the past few decades and now represents up to 25% of the population in developed countries [1]. Obesity and weight gain significantly increase the risk of diabetes and cardiovascular disease (CVD). Obesity can be defined using different approaches; the simplest measures include but are not limited to body mass index (BMI) [2], waist circumference (WC) and waist to hip ratio (WHR). Measures such as WC and WHR are better able to inform on body fat distribution such as abdominal fat; WC in particular correlates well with computed tomography (CT) or dual energy X-ray absorptiometry (DXA) that reflect intraperitoneal adiposity. WC shows stronger associations with risk of type 2 diabetes compared to BMI [3,4]. Comprehensive lipidomic profiling studies in relation to the longitudinal changes in these metrics could enable better understanding of the biological role of circulating lipid species during obesity.

Lipidomics enables the measurement of several hundreds to thousands of molecular lipid species and can facilitate the identification of biomarkers for the assessment of disease risk, including weight gain [5]. High throughput lipidomic analysis in large population cohorts can be useful not only to identify biomarkers but also to better understand the underlying lipid metabolism in obesity and associated comorbidities. Recently, Lamichhane and colleagues have demonstrated the relationship of plasma lipid species with weight gain in patients with psychosis [6]. In addition, a study on the metabolic signature of obesity has highlighted that the human metabolome is a stronger predictor of metabolic health compared to genetic estimates of obesity and BMI [7].

Traditional clinical chemistry approaches or nuclear magnetic resonance (NMR) technologies have been used in a number of studies to define the associations between metabolite levels and prospective changes in measures of adiposity [8,9,10]. However, few studies have reported the association of molecular lipid species with changes in BMI [10,11]. These reports were based on small sample sizes and limited coverage of the lipidome and have not adequately defined how baseline lipidomic measures relate to changes in adiposity over time.

The aim of this study was therefore to perform comprehensive lipidomic analysis and examine the relationship between baseline lipid species levels and the 5-year change in WC and BMI utilizing a large population cohort: the Australian Diabetes, Obesity and Lifestyle Study (AusDiab). Further, we aim to derive metabolic risk scores using linear models to identify those individuals who are at a greater risk of gaining weight in the next 5 years.

## 2. Results 

### 2.1. Characteristics of Participants

The participants’ characteristics at baseline (1999–2000) and changes in obesity metrics are shown in Table 1. The overall mean annual BMI change was 0.16 kg/m^2^/year (95% CI = 0.15–0.17) and that of WC was 0.43 cm/year (95% CI = 0.40–0.46). The annual change in BMI for women was 0.18 kg/m^2^/year and 0.14 kg/m^2^/year for men, *p*-value for the difference = 3.0 × 10^−4^. On average, WC increased by 0.51 cm/year and 0.33 cm/year, in men and women, respectively (*p*-value for the difference = 3.54 × 10^−8^). The mean annual increase in BMI (Figure S1, Table 1) and WC differed by age group (Figure S1). At baseline, 3292 (56.3%), 1265 (21.6%), 592 (10.1%) and 1802 (30.8%) had high cholesterol, high triglyceride, low HDL-C and hypertension, respectively. We note that as the associations shown in Table 1 are not adjusted for covariates some findings can appear counterintuitive; for example, people with a lower energy intake have a greater increase in WC. This group contains a greater proportion of people with small WC and so despite a smaller energy intake still show a greater increase in WC. Similarly, people with shorter TV viewing time also show a greater increase in WC, however this does not consider that this group may also have higher energy intake of other risk factors. Thus, these figures should be interpreted with caution.

### 2.2. Association of Lipid Species with Change in WC

**Figure 1:**
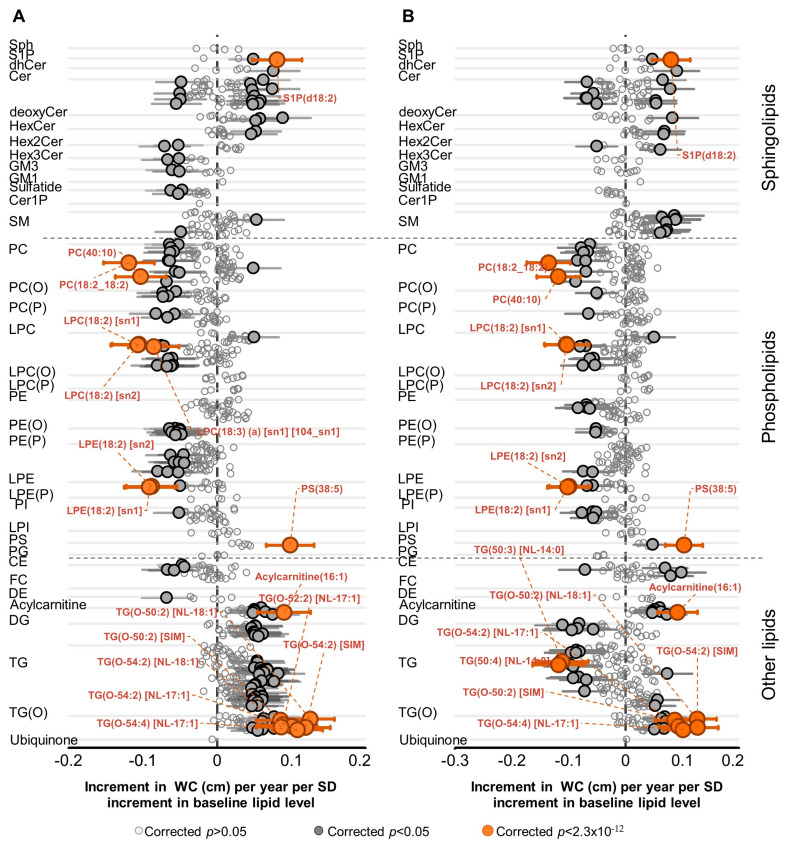
Association of lipid species with change in waist circumference: linear regression analyses of SD normalized lipid species against annualized change in waist circumference were performed; (**A**) adjusting for baseline age, sex and baseline WC or (**B**) for baseline age, sex, baseline WC, total cholesterol, HDL-C, triglycerides, smoking status, education, exercise time and television viewing time. The β-coefficients (95% CIs) represent the change in WC per year associated with a SD difference of the lipid species at baseline. Open circles show lipid species with corrected *p* > 0.05, closed circles show corrected *p* < 0.05 and orange circles show lipid species with the lowest corrected *p*-values. Whiskers represent the 95% confidence intervals. Abbreviations: CE, cholesteryl ester; Cer, ceramide; Cer-1-P, ceramide-1-phosphate; DE, dehydrocholesterol; deoxyCer, deoxyceramide; DG, diacylglycerol; dhCer, dihydroceramide; FC, free cholesterol; GM1, GM1 ganglioside; GM3, GM3 ganglioside; HDL-C,high-density lipoprotein cholesterol; HexCer, monohexosylceramide; Hex2Cer, dihexosylceramide; Hex3Cer, trihexosylceramide; LPC, lysophosphatidylcholine; LPC(O), lysoalkylphosphatidylcholine; LPC(P), lysoalkenylphosphatidylcholine; LPE, lysophosphatidylethanolamine; LPE(P), lysoalkenylphosphatidylethanolamine; LPI, lysophosphatidylinositol; NL, neutral loss; PC, phosphatidylcholine; PC(O), alkylphosphatidylcholine; PC(P), alkenylphosphatidylcholine; PE, phosphatidylethanolamine; PE(O), alkylphosphatidylethanolamine; PE(P), alkenylphosphatidylethanolamine; PG, phosphatidylglycerol; PI, phosphatidylinositol; SM, sphingomyelin; sn, stereospecifically numbered; Sph, sphingosine; S-1-P, sphingosine-1-phosphate; TG, triacylglycerol; TG(O), alkyl-diacylglycerol.

There were 134 lipid species (excluding TG measures using single ion monitoring) associated with WC change after adjusting for baseline age, sex and WC in the whole cohort. These include species of acylcarnitine (7), diacylglycerol (8), lysophosphatidylcholine (12), phosphatidylcholine (13), triacylglycerol (29 including SIMs) and alkyl-diacylglycerol (14 including SIMs) (Figure 1A, Table S3). Glycerophospholipid species containing 18:2 or 18:3 fatty acids typically showed a strong negative association with increasing WC (i.e., lower baseline levels predicted an increase in WC) (Figure 1A,B). The species positively associated with WC change were dominantly acylcarnitine and glycerolipids (diacylglycerol, triacylglycerol and alkyl-diacylglycerol species) with TG(O-50:2) [NL-18:1] representing the most significant species (0.125 cm increment in WC per year per SD increment in baseline lipid level, *p* = 2.8 × 10^−11^). Additional adjustment for total cholesterol, HDL-C, triglycerides, exercise time, educational attainment, smoking and television viewing time resulted in fewer significant lipid species and lowering of effect sizes (Figure 1B, Table S3). While some phospholipid and most TG and DG signatures were lost upon adjustment for clinical lipids, the TG(O) and the 18:2 fatty acid profile were not substantially modified (Figure 1B, Table S3). As a sanity check we repeated the analyses excluding people on lipid lowering drugs and demonstrated that the use of hypolipidemic drugs such as statins at baseline had minimal effect on the lipidomic associations with longitudinal changes in WC. The correlation between beta-coefficients before and after excluding individuals on stain treatment was very high (r^2^ = 0.98).

The baseline metabolic phenotype associated with increasing WC appear to be independent of baseline dietary intake, i.e., the influence of baseline diet on the associations of baseline lipid species with 5-year change in WC was very small; the correlation between the beta-coefficients of lipids before and after accounting for diet showed an r^2^ = 0.9598 (Figure S2). To exclude the effect of weight loss on the associations between lipid species and weight change, secondary analyses were performed after excluding individuals who lost more than 5% of their WC from baseline (n = 748). We found many significant associations (Table S4, Figure S3); the pattern of which is similar to what was observed in the whole cohort except for a few species. Further to the standard covariates in our models, we also adjusted for FBG and observed no substantial effect on the current analyses. As a further sensitivity analyses we examined the association of lipid species with changes in WC after excluding obese individuals at baseline. However, the pattern of associations remained very similar before and after excluding obese individuals (r^2^ of the plot of beta-coefficients with and without obese individuals = 0.94). Similarly, in analyses excluding individuals with diabetes, we did not observe significant difference to the general population.

The lipidomic signatures associated with change in WC and the signature associated with the baseline WC were not highly correlated (r^2^ = 0.163) (Figure S4). Generally, the lipidomic associations with baseline WC were stronger and span across all lipid classes/subclasses with 561 species significantly associated with baseline WC (Table S5) compared to associations with change in WC. 

### 2.3. Association of Lipid Species with Change in BMI

**Figure 2:**
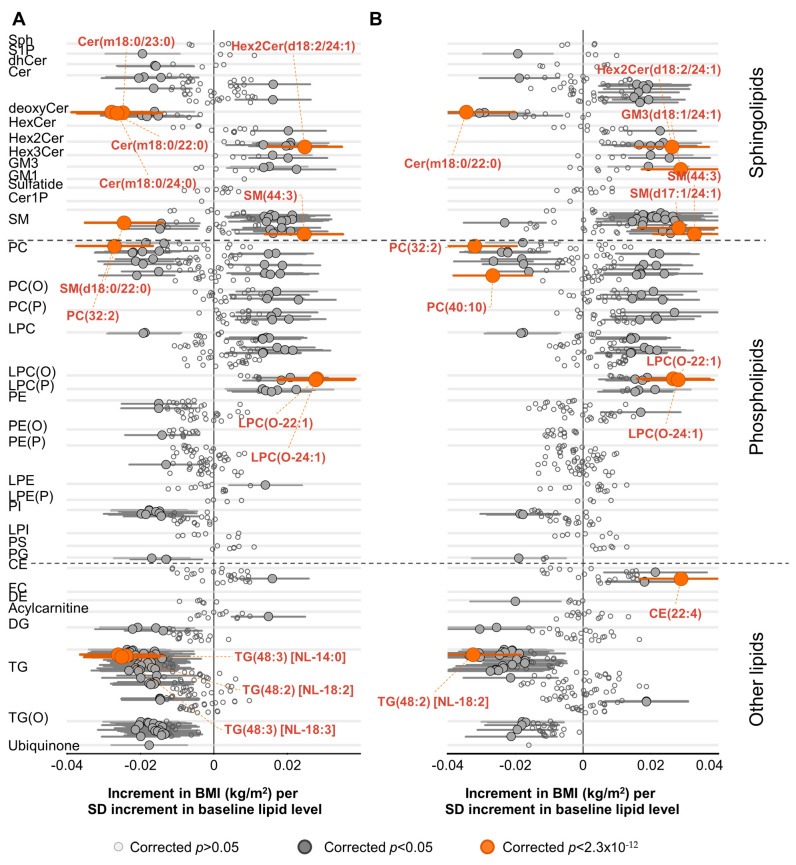
Association of lipid species with change in BMI. Linear regression analyses of SD normalized lipid species against annualized change in BMI were performed adjusting for (**A**) baseline age, sex and baseline BMI or (**B**) for baseline age, sex, baseline BMI, total cholesterol, HDL-C, triglycerides, education, smoking status, exercise time and television viewing time (panel B). The β-coefficients (95% CIs) represent the change in BMI per year associated with a SD difference of the lipid species level at baseline. Open circles show lipid species with corrected *p* > 0.05, closed circles show corrected *p* < 0.05 and orange circles show lipid species with the lowest corrected *p*-values. Whiskers represent the 95% confidence intervals. Abbreviations: CE, cholesteryl ester; Cer, ceramide; Cer-1-P, ceramide-1-phosphate; DE, dehydrocholesterol; de-oxyCer, deoxyceramide; DG, diacylglycerol; dhCer, dihydroceramide; FC, free cholesterol; GM1, GM1 ganglioside; GM3, GM3 ganglioside; HDL-C,high-density lipoprotein cholesterol; HexCer, monohexosylceramide; Hex2Cer, di-hexosylceramide; Hex3Cer, trihexosylceramide; LPC, lysophosphatidylcholine; LPC(O), lysoalkylphosphatidylcho-line; LPC(P), lysoalkenylphosphatidylcholine; LPE, lysophosphatidylethanolamine; LPE(P), lysoalkenylphosphati-dylethanolamine; LPI, lysophosphatidylinositol; NL, neutral loss; PC, phosphatidylcholine; PC(O), alkylphosphati-dylcholine; PC(P), alkenylphosphatidylcholine; PE, phosphatidylethanolamine; PE(O), alkylphosphatidylethanola-mine; PE(P), alkenylphosphatidylethanolamine; PG, phosphatidylglycerol; PI, phosphatidylinositol; SM, sphingo-myelin; Sph, sphingosine; S-1-P, sphingosine-1-phosphate; TG, triacylglycerol; TG(O), alkyl-diacylglycerol.

After adjusting for baseline age, sex and BMI, there were 63 lipid species positively associated with change in BMI and 93 lipid species showing a negative association. (Figure 2A, Table S6). LPC(O-22:1) displayed the strongest association (0.027 kg/m^2^ increase per year per SD increment in baseline lipid level, corrected *p*-value = 7.7 × 10^−5^) (Figure 2A, Table S6). Strong associations were observed with species of triacylglycerol, deoxyceramide and several phospholipid classes. A model adjusted for age, sex, BMI, total cholesterol, HDL-C, triglycerides, smoking status, education, physical activity time and TV viewing time resulted in relatively weaker associations (Figure 2B). A further adjustment for baseline dietary intake (including daily total energy, dairy, saturated fat, fiber and protein intake) did not materially change the pattern. The cross-sectional associations of the lipidome with baseline BMI were different from associations with changes in BMI over time (Figure S5, Table S7). 

### 2.4. Overlapping Associations of Lipid Species with Change in WC and Change in BMI

**Figure 3:**
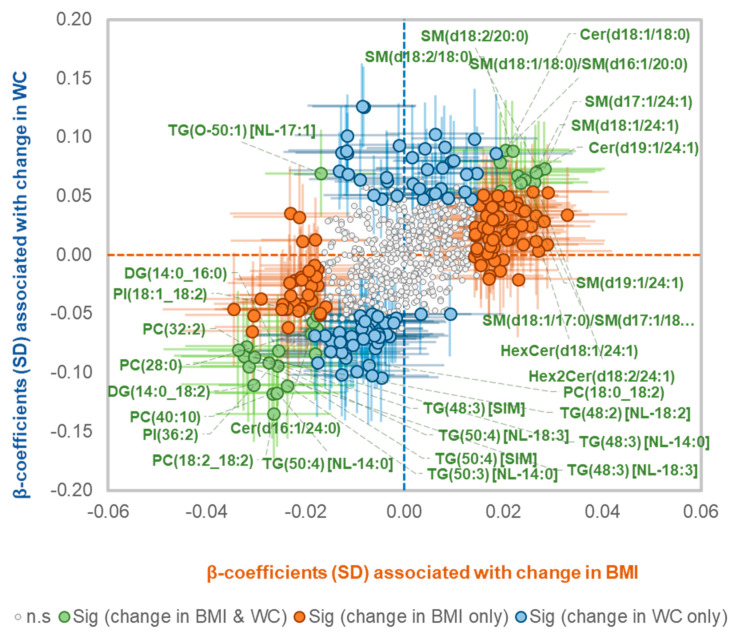
Association of lipid species with change in waist circumference and BMI. Linear regression analyses of SD normalized lipid species concentrations against annualized change in WC or BMI were performed, adjusting for baseline age, sex, baseline WC/BMI, total cholesterol, HDL-C, triglycerides, education, smoking status, exercise time and television viewing time. The β-coefficients of the associations with change in BMI were plotted against the β-coefficients of the associations with change in WC. β-coefficients for lipid species that were associated with both a change in WC and change in BMI are shown in green. β-coefficients for lipid species that were associated only with a change in BMI are orange and only with a change in WC are blue. The whiskers represent 95% confidence intervals.

Change in BMI and change in WC over follow up were significantly correlated (r^2^ = 0.48, *p* < 0.0001) (Figure S6). We assessed whether the pattern of association between lipid species and change in WC was different from that with change in BMI. In age, sex and baseline WC or BMI adjusted models, 22 species were associated with both change in BMI and WC; 8 TG(O) species (enriched for O-18:1 alkyl chains, identified as [NL-17:1] species in Table S3) in opposite direction and the rest in same direction (Figure S7). In total, 30 lipid species were associated with both change in WC and BMI in age, sex, baseline BMI or WC plus other risk factors adjusted model (Figure 3) excluding lipid measurements based on SIMs which represent composite measures. While lipid species, particularly those containing the 18:2 fatty acid, were more strongly associated with changes in WC than with changes in BMI (Tables S3 and S6, Figure 3), the odd and branched chain fatty acid containing lipid species were associated mainly with change in BMI. The associations of lipid species with changes in WC were correlated with the associations with changes in BMI as shown in Figure 3 (r^2^ = 0.3).

**Figure 4:**
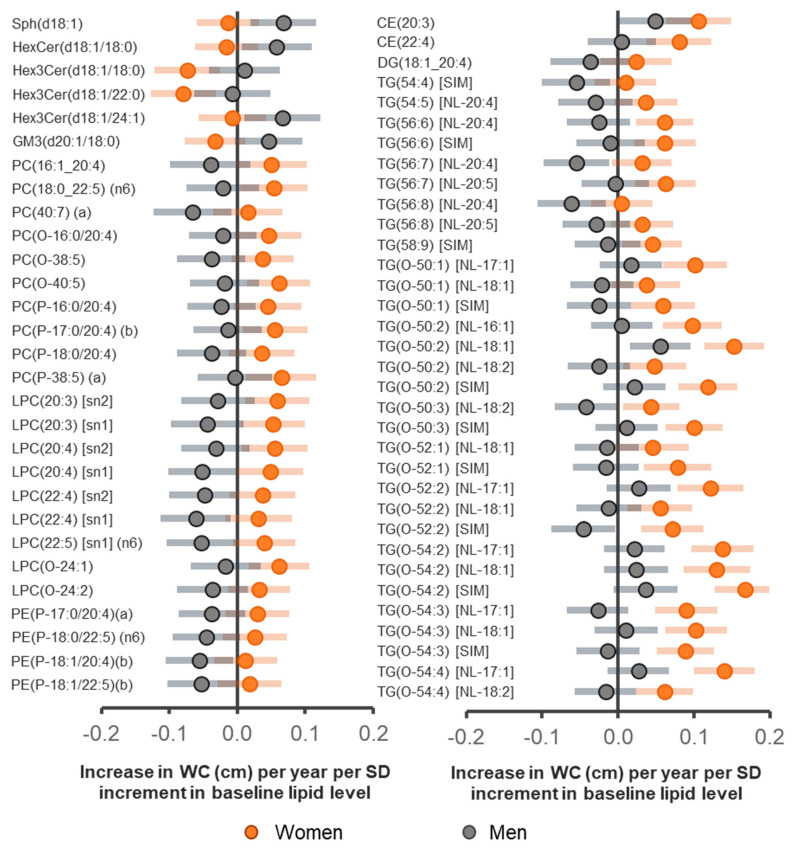
The interaction of sex in the associations of lipid species with change in WC. Linear regression analyses of lipid species with change in WC were performed in models adjusting for age, sex, baseline WC, cholesterol, HDL-C, triglycerides, smoking, education, physical exercise time and TV viewing time including an interaction term for sex. The change in WC (an outcome) was annualized and each lipid (a predictor) was scaled to unit SD prior analysis. The β- coefficients for each sex where the associations with change in WC showed a significant interaction (interaction *p* < 0.05) are plotted. Gray circles (for men) and orange (for women) represent 1 cm increase in WC per year per SD increase in the lipid predictor. Error bars show 95% confidence intervals.

The associations between baseline lipidomic profiles and change in WC were sex-specific. A total of 54 lipid species (excluding TG and TG(O) species monitored by SIMs) showed a nominally significant interaction with sex in predicting change in WC (Table S8). All TG(O) species showed a positive association with change in WC in women, while no species showed a significant association in men. Certain phospholipids and ether-linked phospholipid species enriched in the 20:4 fatty acid tend to be negatively associated with change in WC in men but positively associated in women (Figure 4, Table S8). In contrast to WC, only 25 lipid species showed a nominal sex interaction in the association with change in BMI after controlling for age, sex, baseline BMI, total cholesterol, HDL-C, triglycerides, education, smoking status, exercise time and television viewing time (Table S9).

### 2.5. Multivariate Modeling to Predict Change in Waist Circumference

We developed multivariate models to generate scores for change in WC and subsequently predicted the risk of gaining waist circumference (>5% increase from baseline) during the 5-year follow-up time. The scores for change in WC were derived either from: (1) a base model including baseline age, sex, WC, total cholesterol, HDL-C, triglycerides, education, smoking, exercise time, TV viewing time and baseline energy intake (Model 1) or (2) Model 1 plus a lipidomic score derived from the lipid species associated with changes in WC in the univariate analyses (Model 2). In a sex stratified analysis, using Model 1, the risk of gaining WC by more than 5% was 2.1 times higher among men in the top quintile relative to those in the bottom quintile (odds ratio, 95% CI = 2.1, 1.6–2.8). Based on the Model 2, the risk of >5% WC gain in the Q5 relative to Q1 was 2.3 times higher (Odds ratio, 95% CI = 2.3, 1.7–3.0) which was comparable to the base model (Table 2). A likelihood ratio test showed a significant improvement in the performance of Model 2 (AIC = 3242.4) relative to Model 1 (AIC = 3248.9) to predict waist gain among men (*p*-value for likelihood ratio test = 3.53 × 10^−3^).

**Figure 5:**
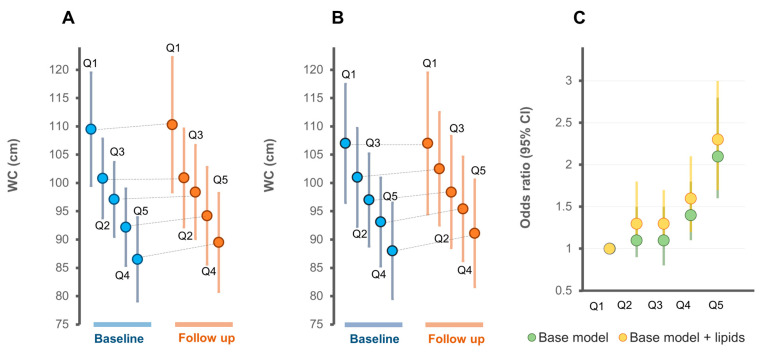
Risk of gaining WC across quintiles of metabolic scores in men. (**A**) Baseline and follow-up WC measures (mean ± SD) across quintiles of the metabolic score derived from the base model, Model 1 and (**B**) from the base model plus lipids, Model 2; (**C**) the relative risk of a >5% WC change across quintiles of the metabolic score. Base model, age, sex, WC, total cholesterol, HDL-C, triglycerides, smoking, exercise time, TV viewing time, energy intake; lipids, all the lipid species associated with change in WC.

Among men, the WC trajectory across the quintiles derived from Model 1 (Figure 5A) and Model 2 (Figure 5B) were only slightly different. The risk of gaining >5% of WC was greater among the four quintiles (Q2 to Q5) relative to Q1 in Model 2 compared to the Model 1 (Figure 5C). 

In women, based on Model 1, the risk of >5% WC change in Q2–Q5 relative to Q1 was consistently higher relative to men. The addition of lipidomic score to the base model particularly improved the risk of gaining WC among Q5 to Q1 (odds ratio, 95% CI = 5.4, 3.8–6.6) (Table 3). 

**Figure 6:**
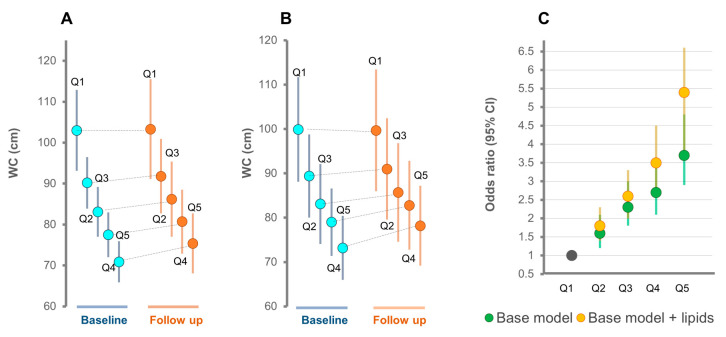
Risk of gaining WC across quintiles of metabolic scores in women. (**A**) Baseline and follow-up WC measures (mean ± SD) across quintiles of the metabolic score derived from the base model, Model 1, and (**B**) from the base model plus lipids, Model 2. (**C**) The relative risk of a >5% WC change across quintiles of the metabolic score. Base model (age, sex, WC, total cholesterol, HDL-C, triglycerides, smoking, exercise time, TV viewing time, energy intake); lipids, all the lipid species associated with change in WC.

Overall, Model 2 (AIC = 4283.1) performed better than Model 1 (AIC = 4347.7) to predict WC gain among women (*p*-value for likelihood ratio test =3.4 × 10^−16^). The WC trajectories across the quintiles derived from Model 1 (Figure 6A) and Model 2 (Figure 6B) for women show similar pattern as for men but magnitudes were different. Model 2 compared to Model 1 identified a higher proportion of individuals with >5% WC change across quintiles of the metabolic score (Figure 6C). 

In an analysis, combining men and women together, the addition of lipid species to the base model captured a larger proportion of individuals with the risk of gaining >5% WC particularly in the top quintile. We observed a 3.5-fold higher risk (Q5 vs. Q1) using Model 2 compared to only a 2.9-fold increased risk using the Model 1 (Table S10, Figure S8). Model 2 (AIC = 7294.6) relative to Model 1 (AIC = 7370.8) showed a significant improvement in performance in predicting WC gain in the whole cohort (*p*-value for likelihood ratio test = 1.9 × 10^−15^).

Examining the baseline and follow-up WC by quintiles, we observed a wide spread of WC between quintiles in Model 1 (Figure S8A). In contrast there is a narrower range in the WC measure between quintiles when lipid species were included in the model (Model 2, Figure S8B). The odds of gaining >5% of WC was greater in Q2 to Q5 relative to Q1 in Model 2 (containing lipid species) compared to the base model with no lipid species (Model 1, Table S10 and Figure S8C). 

## 3. Discussion

Perturbations across multiple lipid classes were associated with change in WC and change in BMI over five years. Many of these lipid classes and subclasses have been associated with cardiometabolic disorders such as obesity [7,12,13,14] and diabetes [13,15,16,17]. However, the profiles associated with change in WC and BMI did not resemble the profiles associated with baseline WC and BMI. The difference in cross-sectional associations of lipids with WC or BMI and associations with changes in WC or BMI is not surprising, as firstly we adjusted for baseline BMI or WC when calculating associations with change in WC and BMI and secondly, people who are lean at baseline are more likely to gain weight compared to those who already have high BMI or WC [17]. We also observed that the baseline lipidomic profiles associated with change in WC or BMI were sex-dependent. Some of the lipid species (particularly in the alkyl-diacylglycerol class) showed contrasting associations in men and women with WC change and change in BMI, although the direction of association of most lipid species was common. Importantly, we show that the lipidomic profile associated with change in BMI or WC was independent of baseline dietary intake. Overall, our findings highlight the potential biological role of lipids in weight/WC gain and suggest that the risk of increasing WC or BMI in the general population might be predicted using baseline lipidomic profiles. 

In the AusDiab cohort, it has been reported that weight gain over 5 years is more likely in those who are lean/young individuals at baseline than obese/older. Consequently, the baseline age, WC and/or BMI are among the major predictors [18]. It is also likely that diet and food choice pattern influence weight gain. Of note, Pearcey et al. have reported that weight gaining people consume more carbohydrate and fat and larger meal sizes [18]. Low omega-6 to omega-3 ratio and inflammatory white blood cells as well as reduced vascular endothelial growth factor has been associated with Mediterranean diet that protect from weight gain [19]. Certain diets also appear to alter lipid levels. For instance, sugar sweetened beverage consumption increases circulating ceramides [20]; increase in saturated fatty acid is associated with a decrease in triglycerides [21]. Indeed, lipid metabolism plays its role in weight gain. Abnormal lipid metabolism (such as insufficient basal lipolysis characterized by high basal/low hormone stimulated lipolysis) predicted weight gain in women [22]. We have previously shown that the plasma lipidome is strongly associated with age and BMI and sex with interaction effects in the association of lipid species with obesity [23]. Thus, the baseline lipidomic profile associated with the risk of WC or weight gain could be a reflection of the interaction between baseline dietary intake, biological factors (baseline age, sex and adiposity) and possibly other factors. 

The microbiome is another contributing factor to the pathophysiology of weight change and potentially confounds the observed associations of lipids and risk of weight or WC gain. Although not yet clearly defined, there is a strong and dynamic relationship between the gut microbiota and obesity. The microbiome influences metabolism of certain substrates; microbiome in obese mice could harvest more energy from food than their lean counterparts and subsequently, colonizing lean mice with microbiota from obese mice significantly increased total body fat suggesting that the gut microbiome plays an important role in obesity [24]. Transplantation of microbiota can enhance energy release from plant foods but also modulate genes associated with energy deposition such as fasting-induced adipocyte factor (Fiaf), a circulating lipoprotein lipase inhibitor [25]. In contrast to mice with microbiota, those mice reared in a germ-free environment were protected from high fat diet induced obesity [26]. Indeed, distinct alterations in the metabolome were observed among a high-fat induced obesity mouse model in relation to the host–microbial nutritional adaptation [26]. Overall, these findings suggest the role of microbiota in modulating host metabolism and the pathophysiology of obesity. The plasma lipidomic profiles associated with increased WC or BMI measured in this study have been adjusted for baseline age, sex, BMI, clinical lipids and other lifestyle measures and so likely reflect the basal metabolic state of the individuals, independent of these factors but capturing the complex interaction effects as well as microbiome contributions and other environmental and genetic effects. 

Several phosphatidylcholine species, particularly those containing linoleic acid (18:2) such as PC(18:0_18:2), PC(18:2_18:2) and PC(18:2_20:5) were negatively associated with both change in BMI and WC, suggesting a common mechanism. Indeed, circulating linoleic acid (LA) levels have been shown to be inversely associated with type 2 diabetes driven particularly by impaired post-load glucose [27,28]. LA is an essential fatty acid completely derived from diet (such as vegetable oils). Upon dietary intake LA can be esterified to form lipids such as triacylglycerols, cholesteryl esters and phospholipids. Although we have no dietary information for LA in our cohort, adjusting out for common dietary variables such as energy, fat and protein did not affect the observed association of LA with change in obesity. While the negative association of LA with change in obesity suggests a protective role against waist or BMI gain, a causal relationship (if any) between plasma LA levels and change in weight/WC cannot be established based on the current findings due to the complex heterogeneity and confounding present in human diets. This will require testing in clinical trials in order to make dietary recommendations. 

Medium to long chain acylcarnitine species including acylcarnitine (12:0), acylcarnitine (16:1) and acylcarnitine (18:1) were positively associated with changes in adiposity (mainly with changes in WC). This suggests not only the potential role of acylcarnitine species as biomarkers for predicting future weight gain but also as drivers of changes in obesity. The altered acylcarnitine species with changes in WC could be related to mitochondrial function. One of the main functions of mitochondria is to produce adenosine triphosphate (ATP) from acetyl-CoA (via β-oxidation). Development of obesity has been shown to be associated with impaired β-oxidation and reduced mitochondrial biogenesis [29]. At the same time a reduction in mitochondrial size and respiratory chain activity have been reported in weight gain [30]. Using metabolomics, Kang et al. have demonstrated a significant increase in plasma acylcarnitines in overweight subjects [31]. Thus, the imbalance in mitochondrial fatty acid oxidation resulting in altered levels of acylcarnitines appear to precede changes in adiposity. Indeed, altered acylcarnitine metabolism (i.e., increased levels) have been implicated in insulin resistance and type 2 diabetes [32,33,34]. Whether the altered mitochondrial function or the acylcarnitines themselves are causal for the long term weight gain remains to be determined. 

A further novel observation in this study was that the baseline plasma alkyl-diacylglycerol levels and some ether lipids strongly predicted future waist gain, especially in women. Alkyl-diacylglycerols contain an alkyl group and two acyl chains attached to a glycerol backbone. Alkylglycerols are ether lipids mostly found in high abundance in shark liver oil [35], but in humans, they have not been well characterized. Studies show that alkylglycerols exhibit anti-diabetic and anti-inflammatory properties [36,37]. However, data on the role of alky-diacylglycerols in weight gain and obesity are lacking. Ether lipids in general are derived from peroxisomes that are implicated in the regulation of adipocyte thermogenesis and dynamics [38]. Peroxisomes are particularly abundant in brown adipose tissue (BAT) relative to white adipose tissue (WAT) and alkylglycerols have been shown to maintain beige adipose tissue in early life in a mouse model [39]. It has been shown that there is a sex difference in the abundance of BAT (higher in women compared to men) [40] and women had a higher WC increase compared to men. Thus, it could be the interplay between peroxisomal dynamics associated with altered ether lipid metabolism (presumably in abdominal fat) that is driving changes in WC in women. The strong positive association of alkyl-diacylglycerol (mainly the O-18:1 species) with change in WC in women may indicate a differential metabolic regulation of these lipids (O-18:1 alkyl-diacylglycerols) with WC change according to sex. 

We observed sex-specificity of the association of many lipid species with change in WC and BMI. Age and sex-specificity in the association of lipidome with changes in obesity had not been previously documented. However, sex-specificity in the association of free fatty acids with insulin resistance [41] and phospholipids with metabolic syndrome have been reported [42,43]. Further to these reports, a recent study identified sex differences of metabolic profiles in BAT and WAT [44]. There is a growing interest in sex-dimorphism particularly for those disorders associated with metabolic perturbations. There is a sex-specificity in the risk of CVD [45,46] and lipid metabolism in obesity [43,47]. Such differences in disease risk are potentially reflected in metabolic profiles. The underlying mechanism for sex-specificity in the association of lipids with WC or BMI change observed here is not clear but it is possible that the endocrine system and genetics underpin these differences. Generally, younger participants, particularly women, are more likely to gain WC and BMI over time [19]. Thus, it is likely that the underlying metabolic state associated with changes in obesity in men and women or in younger compared to older people are different. 

Multivariate models developed to generate risk scores for change in WC over a 5-year follow-up showed that the addition of lipidomic score derived from lipid species (comprising of a mixture of sphingolipid, phospholipid, glycerolipid and other lipids) to the base model improved the stratification of individuals at high and low risk of gaining >5% WC. Our modeling suggests that the base model primarily captures the effect of age, sex and baseline WC on the risk of WC change, while the model containing lipid species captures the metabolic disturbance associated with weight gain. A logistic regression analysis between quintiles of risk revealed that the addition of lipid species to the traditional risk factors identified individuals in the top quintile with higher odds of gaining WC. In an earlier study in the European Prospective Investigation into Cancer and Nutrition (EPIC) study a C-statistic of only 0.57 was reported using risk models with common cardiometabolic risk factors to predict a substantial weight gain (as defined by more than 10% increase from baseline during 5 year follow up) [48]. However, the EPIC study lacked the metabolic profile component that would have improved the predictive power of the model. Weight or waist gain are highly dynamic and complex processes driven by genetics, environment, dietary and life style factors. The human lipidome captures aspects of all these factors and therefore may be useful to identify those individuals at greater risk of waist gain to aid clinicians to make informed early decisions on appropriate interventions. Indeed, WC has become a vital marker in clinical practice and is an important target for reducing adverse health outcomes [49,50]. 

The strengths of this study include the large, national, population-based sample which facilitates the generalizability of our findings. In addition to this, the plasma lipidome coverage was broad (measuring over 700 lipid species across 36 lipid classes/subclass) using the state-of-the-art LC-MS/MS technology. The potential limitations include lack of a similar population-based cohort for replication of our findings. Moreover, low initial response rate and modest loss to follow up in the AusDiab study may have led to a selection bias. Another limitation is the ethnicity of the present study population, which might be over-represented by white or Europid origin and this may limit the generalizability of the findings to other populations. Nevertheless, this study is the first of its kind to explore the potential of baseline lipidomic profiles to define and predict future gain in measures of obesity (WC and BMI) and to further explore whether these are sex-specific. 

## 4. Materials and Methods

### 4.1. Study Design and Participants

The AusDiab is a population-based cohort study of diabetes and associated risk factors on Australian adults. During the baseline survey conducted in 1999/2000, 11,247 subjects who were ≥25 years were recruited [13]. In the subsequent follow-up during 2004/2005, 6400 participants were re-examined [14]. In this analysis, of the 10,358 subjects who had a baseline fasting plasma sample, 4509 were excluded due to (1) being lost to follow-up (n = 4458); (2) having insufficient amount of plasma samples for analysis (n = 13); (3) technical issues during MS analysis (n = 19). Thus, 5849 subjects who had complete data at baseline and at the 5-year follow-up were included. Annual change in each metric (BMI or WC) was calculated as the difference in the metric between the baseline and 5-year follow-up divided by the follow-up duration in years. 

### 4.2. Data Collection and Laboratory Measurements

The collection of demographic and behavioral data of the participants has been described in detail elsewhere [11,51]. Fasting blood samples were taken and BMI, weight and WC were measured at baseline and follow-up [51]. Collection of physical activity levels [52] and TV viewing time [53] have been described previously. The categorization of smoking status (smokers (current), non-smokers (never smoked) and (ex-smokers)) and daily energy intake (kJ/day) have also been described [54]. Fasting plasma cholesterol and lipoprotein concentration including total cholesterol, high density cholesterol, (HDL-C), low density lipoprotein cholesterol (LDL-C) and triglycerides, fasting plasma glucose (FPG) and 2 h post load glucose (2h-PLG) were measured using standard protocols [55]. Dietary information was collected using a validated food frequency questionnaire (FFQ) [11]. The study was approved by the Human Research Ethics Committee at the Alfred Hospital, Melbourne, Australia.

### 4.3. Ethics

This study used a dataset from the AusDiab biobank (project grant APP1101320) approved by the Alfred Human Research Ethics Committee, Melbourne, Australia (project approval number, 41/18).

### 4.4. Plasma Lipidomic Profiling

#### 4.4.1. Lipid Extraction

Plasma lipids were extracted as described previously [56] but were assisted by an automated liquid handling robot (MicroLAB STAR, Hamilton, Biosystems, Inc., Reno, NV, USA). In brief, 10 μL of plasma was mixed with 100 μL of butanol:methanol (1:1) containing 10 mM ammonium formate in Eppendorf tubes. Internal standards (22 in total) representing major lipid classes (Table S1) were included in the extraction mix. Samples were vortexed thoroughly and sonicated in a sonicator bath for 1 h maintained at (<20 °C) and then centrifuged (14,000× *g*, 10 min, 20 °C). A 100 μL aliquot of the lipid extract was then transferred into 2 mL vials with glass inserts.

#### 4.4.2. Liquid Chromatography Tandem Mass Spectrometry (LC-MS/MS)

Lipid analysis was performed by LC ESI-MS/MS using a triple quadruple mass spectrometer (Agilent 6490 QQQ mass spectrometer with an Agilent 1290 series HPLC system and a ZORBAX eclipse plus C18 column (2.1 × 100 mm × 1.8 mm). Mass spectrometry analysis was performed in a positive ion mode with dynamic scheduled multiple reaction monitoring (MRM) as described previously [57]. There were however, some modifications to our previous method; we utilized a dual column setup (in which one of the column is set to equilibrate while the other is running a sample). The temperature was reduced to 45 °C from 60 °C with modifications to the chromatography to enable similar level of separation as in previous method, starting at 15% solvent B and increasing to 50% B over 2.5 min, then quickly ramping to 57% B for 0.1 min. For 6.4 min, %B was increased to 70%, then increased to 93% over 0.1 min and increased to 96% over 1.9 min. The gradient was quickly ramped up to 100% B for 0.1 min and held at 100% B for a further 0.9 min. This was a total run time of 12 min. The column was then brought back down to 15% B for 0.2 min and held for another 0.7 min prior to switching to the alternate column for running the next sample. The column that was being equilibrated was run as follows: 0.9 min of 15% B, 0.1 min increase to 100% B and held for 5 min, decreasing back to 15% B over 0.1 min and held until it was switched for the next sample. We used a 1 μL injection per sample and the following mass spectrometer conditions were used: gas temperature, 150 °C; gas flow rate, 17 L/min; nebulizer, 20 psi; sheath gas temperature, 200 °C; capillary voltage, 3500 V and sheath gas flow, 10 L/min. The detailed mass spectrometry conditions are presented in the supplementary file (Table S1). Given the large sample size, samples were run across several batches, as described above. Quality control samples; Plasma Quality Control samples (PQC) and Technical Quality Control samples (TQC), were included in the run to assess the assay performance. 

#### 4.4.3. Lipid Classes/Subclasses and Species 

A total of 706 distinct molecular lipid species excluding SIMs across 36 classes/subclasses, representing sphingolipids, glycerophospholipids, glycerolipids and sterols were measured (Table S2). The number of lipid species in each class/subclass ranged from 1 free cholesterol (FC) to 77 triacylglycerol (TG) species. 

#### 4.4.4. Data Processing 

The chromatographic peaks were integrated using the Mass Hunter (B.07.00, Agilent Technologies, Santa Clara, CA, USA) software and assigned to a specific lipid species based on MRM (precursor/product) ion pairs and retention time. The relative concentration of each lipid species was determined by comparing their peak areas to the relevant internal standard (Table S1). Previously generated response factors for each species were used [58]. Lipid data were log10 transformed and standardized to unit standard deviation and 0 mean prior to analysis. The AusDiab study was run in multiple batches (27 in total). Batch effects were corrected using a median centering approach utilizing PQC samples. Over 90% of the lipid species were measured with a coefficient of variation <20% (based on PQC samples). No lipid species were removed on grounds of missing values or high CV. However, outliers deemed to be of technical origin, such as missed injections (n = 19), were excluded from the downstream analysis.

### 4.5. Statistics

#### 4.5.1. Univariate Analyses 

Each species was examined for the association with annualized changes in WC using linear models adjusted for baseline age, sex and WC. Associations with change in BMI were adjusted for baseline age, sex and BMI. Further adjustments for covariates such as total cholesterol, HDL-C, triglycerides, exercise time, smoking status and TV viewing time were also performed. Additional models were adjusted for daily total energy, dairy, saturated fat, fiber and protein intake to observe whether the associations were independent of baseline dietary intake. We also examined if the associations between changes in WC or BMI were sex-specific by testing for sex interactions (i.e., including sex as an interaction term in the models). *p*-values were corrected for multiple comparisons using the Benjamini–Hochberg procedure [59]. A nominal *p*-value of 0.05 was considered as suggestive of an interaction.

#### 4.5.2. Multivariate Modeling 

Lipid concentrations were log10 transformed and scaled to unit standard deviation prior to modeling. We used ridge regression models to generate metabolic scores. A 10-fold cross validation was employed for the generation metabolic scores (i.e., models were generated on the 9/10th of the cohort and used to predict the metabolic score in holdout 1/10th of the cohort). During the model training process, the training set was further 10-fold cross-validated to obtain the lambda value with the lowest mean squared error. This process was iterated so that each sample obtained a metabolic score independent of the model training process. Metabolic scores for change in WC were derived from either traditional clinical risk factors (age, sex, WC, total cholesterol, HDL-C, triglycerides, education, smoking, exercise time, TV viewing time and energy intake) (Model 1) or these factors plus lipidomic score (Model 2). This was performed for men and women separately and for both combined. A lipidomic score was generated for each individual using lipid species associated with changes in WC independent of clinical lipids in the univariate analysis described above. Following the generation of the scores, participants were stratified into quintiles. The relative risk (i.e., the risk of gaining >5% WC from baseline) adjusted for age, sex and baseline WC, was then computed for each quintile. The quality of Model 1 relative to Model 2 was assessed using the Akaike information criterion (AIC) and likelihood ratio test in the lrtest R package. All statistical analyses were performed in R version 3.6.1.

## 5. Conclusions

In conclusion, our study provides comprehensive plasma lipidomic profiles of adiposity allowing the identification of plasma lipidomic signatures of WC or BMI gain in a large population cohort. Sex-stratified analyses showed stronger signatures in women. The observed associations between the baseline lipidomic profile and longitudinal changes in weight and WC shed light on the metabolic basis of obesity progression over time. The distinct nature of the lipidomic profiles that predict change in BMI compared to those that predict change in WC in the present study suggest the metabolic dysregulation preceding each is distinct and further studies may provide insight into the detailed mechanism. Our findings also highlight the potential of basal metabolic levels to predict the risk of weight gain, suggesting that detailed lipidomic investigations in relation to adiposity offer an opportunity to identify biomarkers in clinical research. Finally, we recognize that there is a need to consider the effect of diet, microbiome, genetics and possibly the complex interaction between these and other factors, including interpersonal differences in energy expenditure versus storage, in interpreting lipidomic profiles associated with weight/WC gain. 

## Figures and Tables

**Table 1:** Baseline characteristics of participants associated with changes in WC or BMI.

Characteristics	n	Annualized BMI Change (kg/m^2^) (SD)	^*p* Value	Annualized WC Change (cm) (SD)	^*p* Value
Overall	5849	0.16 (0.41)		0.43 (1.30)	
Sex					
Men	2653	0.14 (0.38)	3.0 × 10^−4^	0.33 (1.51)	3.5 × 10^−8^
Women	3196	0.18 (0.40)		0.51 (1.40)	
Age group					
≥55	2241	0.09 (0.34)	2.2 × 10^−16^	0.29 (1.32)	1.0 × 10^−10^
<55	3608	0.20 (0.41)		0.51 (1.26)	
Education					
High school and below	2178	0.17 (0.37)	7.5 × 10^−1^	0.46 (1.31)	3.9 × 10^−1^
Certificate and diploma	2544	0.16 (0.39)		0.41 (1.30)	
Bachelor’s degree and above	1129	0.16 (0.37)		0.42 (1.27)	
BMI category *					
Normal	2207	0.18 (0.30)	8.2 × 10^−3^	0.49 (1.19)	5.2 × 10^−3^
Overweight	2397	0.15 (0.38)		0.39 (1.29)	
Obese	1245	0.16 (0.52)		0.37 (1.47)	
WC category #					
Low risk	2336	0.18 (0.30)	5.5 × 10^−3^	0.67 (1.12)	6.5 × 10^−34^
Moderate risk	1529	0.15 (0.36)		0.36 (1.23)	
High risk	2015	0.15 (0.49)		0.19 (1.47)	
Smoking					
Current smoker	664	0.22 (0.55)	2.9 × 10^−5^	0.54 (1.39)	8.2 × 10^−4^
Ex-smoker	1694	0.13 (0.39)		0.33 (1.29)	
Non-smoker	3401	0.17 (0.37)		0.45 (1.28)	
TV viewing time (minutes per week)					
Tertile 1 (less than 420)	1960	0.20 (0.36)	8.4 × 10^−7^	0.51 (1.28)	3.0 × 10^−4^
Tertile 2 (420–900)	1938	0.15 (0.37)		0.41 (1.26)	
Tertile 3 (>900)	1929	0.14 (0.42)		0.36 (1.34)	
Diabetes					
Yes	327	0.06 (0.41)	4.7 × 10^−6^	0.33 (1.24)	8.0 × 10^−2^
No	5522	0.17 (0.38)		0.44 (1.30)	
Exercise status based on exercise time (min/week)					
Sedentary (zero min)	909	0.17 (0.41)	5.3 × 10^−1^	0.39 (1.28)	5.5 × 10^−1^
Insufficient (0‒150)	1793	0.17 (0.39)		0.45 (1.29)	
Sufficient (over 150min)	3127	0.16 (0.38)		0.43 (1.27)	
Total energy intake (KJ/day)					
Tertile 1 (<6430.5)	1836	0.17 (0.40)	8.0 × 10^−2^	0.51 (1.35)	4.2 × 10^−4^
Tertile 2 (6430.5–8671)	1901	0.17 (0.39)		0.42 (1.27)	
Tertile 3 (>8671)	1900	0.15 (0.37)		0.36 (1.27)	
Cholesterol (mmol/L)					
High (≥5.5)	3292	0.14 (0.40)	2.4 × 10^−9^	0.36 (1.28)	8.3 × 10^−7^
Low (<5.5)	2589	0.19 (0.40)		0.52 (1.27)	
Triglycerides (mmol/L)					
High (≥2.0)	1265	0.13 (0.40)	6.2 × 10^−5^	0.33 (1.27)	1.7 × 10^−3^
Low (<2.0)	4616	0.18 (0.39)		0.46 (1.31)	
HDL-C (mmol/L)					
High (≥1.0)	5288	0.17 (0.40)	6.7 × 10^−1^	0.44 (1.30)	1.9 × 10^−1^
Low (<1.0)	592	0.16 (0.39)		0.36 (1.26)	
HbA1C (%)					
High (≥6.5)	219	0.06 (0.39)	1.0 × 10^−4^	0.29 (1.19)	9.8 × 10^−2^
Low (<6.5)	5630	0.17 (0.39)		0.43 (1.30)	
HOMA2-B (%)					
Tertile 1 (<110.5)	1743	0.14 (0.34)	6.8 × 10^−1^	0.36 (1.21)	1.5 × 10^−1^
Tertile 2 (110.5–139.4)	1743	0.15 (0.37)		0.43 (1.31)	
Tertile 3 (>139.4)	1743	0.15 (0.41)		0.44 (1.30)	

^*p* values are derived from Student’s t-test for dichotomous characteristics, or one way ANOVA for multiple groups as necessary. Note: the association of baseline characteristics with changes in BMI and WC are not adjusted for any covariates. * BMI category: normal weight, 18.5–24.9 kg/m^2^; overweight, 25–29.9 kg/m^2^; obese: ≥30 kg/m^2^. # WC categories: low risk, <94 cm for men, <80 for women; moderate risk: 94–101.9 for men, 80–87.9 for women; high risk: ≥102 for men, ≥88 for women.

**Table 2:** Multivariate models predicting gain in WC in men.

Quintiles of WC Change Score	Age (Years)	Baseline WC cm Mean (SD)	Follow up WC cm Mean (SD)	Change in WC % (SD)	Change of >5% WC n (Relative Risk)	Risk > 5% WC Change Relative to Q1 (Odds Ratio, 95% CI)
**Model 1 (Base model 1)**						
Q1 (N = 531)	59.5 (12.3)	109.5 (10.0)	110.3 (11.9)	0.7 (5.9)	119 (0.22)	1.0 (reference)
Q2 (N = 531)	55.3 (12.0)	100.8 (7.0)	101.9 (8.7)	1.1 (5.6)	132 (0.25)	1.1 (0.9–1.5)
Q3 (N = 531)	52.3 (11.3)	97.1 (6.6)	98.4 (8.3)	1.4 (5.6)	131 (0.25)	1.1 (0.8–1.5)
Q4 (N = 530)	49.5 (10.6)	92.2 (6.8)	94.2 (8.6)	2.3 (6.2)	152 (0.29)	1.4 (1.1–1.8) *
Q5 (N = 530)	42.4 (10.9)	86.5 (7.4)	89.5 (8.7)	3.3 (6.2)	202 (0.38)	2.1 (1.6–2.8) *
**Model 2 (Model 1 + lipidomic score)**						
Q1 (N = 531)	56.8 (12.2)	107.0 (10.5)	107.0 (12.5)	0.19 (5.6)	110 (0.26)	1.0 (reference)
Q2 (N = 531)	55.0 (12.2)	101.0 (8.7)	102.5 (10.0)	1.2 (5.8)	138 (0.26)	1.3 (1.1–1.8) *
Q3 (N = 531)	53.0 (12.3)	97.0 (8.2)	98.4 (9.9)	1.4 (5.7)	133 (0.25)	1.3 (1.1–1.7) *
Q4 (N = 530)	50.1 (11.8)	93.1 (7.8)	95.4 (9.2)	2.5 (6.1)	157 (0.30)	1.6 (1.2–2.1) *
Q5 (N = 530)	44.1 (11.5)	88.0 (8.5)	91.1 (9.5)	3.6 (6.0)	198 (0.37)	2.3 (1.7–3.0) *

Base model (Model 1): age, sex, WC, total cholesterol, HDL-C, triglycerides, smoking, exercise time, TV viewing time, energy intake. # lipidomic score—scores derived from lipid species associated with change in WC in the univariate analyses. * Significant at *p* < 0.05.

**Table 3:** Multivariate models predicting gain in WC in Women.

Quintiles of WC Change Score	Age (Years)	Baseline WC cm Mean (SD)	Follow up WC cm Mean (SD)	Change in WC (%) Mean (SD)	Change of >5% WC n (Relative Risk)	Risk > 5% WC Change Relative to Q1 (Odds Ratio, 95% CI)
**Model 1 (Base model 1)**						
Q1 (N = 640)	58.6 (12.1)	103.0 (9.9)	103.3 (12.2)	0.3 (7.7)	130 (0.24)	1.0 (reference)
Q2 (N = 639)	55.5 (11.8)	90.2 (6.3)	91.8 (9.1)	1.8 (7.8)	88 (0.25)	1.6 (1.2–2.1) *
Q3 (N = 639)	52.9 (11.2)	83.1 (6.1)	86.2 (9.2)	3.7 (8.2)	157 (0.36)	2.3 (1.8–3.0) *
Q4 (N = 639)	47.7 (10.3)	77.5 (5.5)	80.7 (7.8)	4.3 (8.1)	312 (0.42)	2.7 (2.1–3.4) *
Q5 (N = 639)	41.2 (9.0)	70.9 (5.0)	75.4 (7.3)	6.5 (8.3)	564 (0.50)	3.7 (2.9–4.8) *
**Model 2 (Model 1 + lipidomic score)**						
Q1 (N = 640)	57.8 (11.9)	99.9 (11.8)	99.7 (13.7)	-0.23 (7.6)	131 (0.20)	1.0 (reference)
Q2 (N = 639)	54.0 (12.4)	89.4 (9.4)	91.0 (11.4)	1.8 (7.7)	204 (0.32)	1.8 (1.4–2.3) *
Q3 (N = 639)	51.9 (11.8)	83.1 (9.0)	85.7 (11.1)	3.1 (7.7)	254 (0.40)	2.6 (2.0–3.3) *
Q4 (N = 639)	48.0 (11.2)	79.0 (7.6)	82.8 (10.0)	4.8 (7.9)	301 (0.47)	3.5 (2.7–4.5) *
Q5 (N = 639)	44.2 (10.6)	73.2 (7.2)	78.2 (9.0)	7.2 (8.5)	561 (0.56)	5.4 (3.8–6.6) *

Base model (Model 1): age, sex, WC, total cholesterol, HDL-C, triglycerides, smoking, exercise time, TV viewing time, energy intake. # lipidomic score—scores derived from lipid species associated with change in WC in the univariate analyses. * Significant at *p* < 0.05.

## Data Availability

Because of the participant consent obtained as part of the recruitment process for the Australian Diabetes, Obesity and Lifestyle Study, it is not possible to make these data publicly available. All AusDiab individual level data discussed in the paper can be made available to established researchers by a reasonable written application to the study lead, Professor Jonathan Shaw and the AusDiab Study Committee (Email: jonathan.shaw@baker.edu.au).

## Supplementary Materials

The following are available online at https://www.mdpi.com/article/10.3390/metabo11090646/s1; Table S1: MRM transitions and conditions for examined lipid species, Table S2: Lipid categories, classes/subclasses and number of lipid species measured in this study, Table S3: Baseline lipidomic predictors of changes in waist circumference (WC), Table S4: Baseline lipidomic predictors of changes in WC among waist gainers /sable over 5 years (excluding those subjects who lost over 5% WC from baseline), Table S5: Lipidomic signatures associated with baseline waist circumference, Table S6: Baseline lipidomic predictors of change in BMI, Table S7: Lipidomic signatures associated with baseline BMI, Table S8: Sex-specific associations of baseline lipidomic profile with changes in WC, Table S9: Sex-specific associations of baseline lipidomic profile with changes in BMI, Table S10: Multivariate models predicting changes in WC, Figure S1: Annual waist circumference (WC) change by age and sex. Mean annualized WC change (cm/year) is shown on y-axis for men and women further stratified by age (<55 years and >55 years), Figure S2: The correlation between beta-coefficients of lipids associated with change in WC before and after accounting for diet, Figure S3: Association of lipid species with change in WC, Figure S4: The correlation between beta-coefficients for baseline WC and change in WC, Figure S5: The correlation between beta-coefficients for baseline BMI and change in BMI, Figure S6. The correlation between change in BMI and change in WC, Figure S7. Association of lipid species with change in WC and BMI, Figure S8. Risk of gaining WC across quintiles of metabolic scores.
